# Incidence and predisposing factors for the development of disturbed glucose metabolism and DIabetes mellitus AFter Intensive Care admission: the DIAFIC study

**DOI:** 10.1186/s13054-015-1064-9

**Published:** 2015-10-02

**Authors:** Sofie Van Ackerbroeck, Tom Schepens, Karolien Janssens, Philippe G. Jorens, Walter Verbrugghe, Sandra Collet, Viviane Van Hoof, Luc Van Gaal, Christophe De Block

**Affiliations:** Department of Critical Care Medicine, Antwerp University Hospital, Edegem, Belgium; Faculty of Medicine and Health Sciences, University of Antwerp, Antwerp, Belgium; Department of Endocrinology, Diabetology and Metabolism, Antwerp University Hospital, Wilrijkstraat 10, B-2650 Edegem, Belgium; Department of Clinical Chemistry, Antwerp University Hospital, Edegem, Belgium

## Abstract

**Introduction:**

Elevated blood glucose levels during intensive care unit (ICU) stay, so-called stress hyperglycaemia (SH), is a common finding. Its relation with a future diabetes risk is unclear. Our objective was to determine the incidence of disturbed glucose metabolism (DGM) post ICU admission and to identify predictors for future diabetes risk with a focus on stress hyperglycaemia.

**Methods:**

This single center prospective cohort trial (DIAFIC trial) had a study period between September 2011 and March 2013, with follow-up until December 2013. The setting was a mixed medical/surgical ICU in a tertiary teaching hospital in Belgium. 338 patients without known diabetes mellitus were included for analysis. We assessed the level of glucose metabolism disturbance (as diagnosed with a 75 g oral glucose tolerance test (OGTT) and/or HbA1c level) eight months after ICU admission, and investigated possible predictors including stress hyperglycaemia.

**Results:**

In total 246 patients (73 %) experienced stress hyperglycaemia during the ICU stay. Eight months post-ICU admission, 119 (35 %) subjects had a disturbed glucose metabolism, including 24 (7 %) patients who were diagnosed with diabetes mellitus. A disturbed glucose metabolism tended to be more prevalent in subjects who experienced stress hyperglycaemia during ICU stay as compared to those without stress hyperglycaemia (38 % vs. 28 %, P = 0.065). HbA1c on admission correlated with the degree of stress hyperglycaemia. A diabetes risk score (FINDRISC) (11.0 versus 9.5, P = 0.001), the SAPS3 score (median of 42 in both groups, P = 0.003) and daily caloric intake during ICU stay (197 vs. 222, P = 0.011) were independently associated with a disturbed glucose metabolism.

**Conclusions:**

Stress hyperglycaemia is frequent in non-diabetic patients and predicts a tendency towards disturbances in glucose metabolism and diabetes mellitus. Clinically relevant predictors of elevated risk included a high FINDRISC score and a high SAPS3 score. These predictors can provide an efficient, quick and inexpensive way to identify patients at risk for a disturbed glucose metabolism or diabetes, and could facilitate prevention and early treatment.

**Trial registration:**

At ClinicalTrials.gov NCT02180555. Registered 1 July, 2014.

## Introduction

Stress hyperglycaemia (SH) is reported to occur in 50−85 % of critically ill patients admitted to the intensive care unit (ICU) and is associated with poorer outcome in a variety of clinical settings (e.g., myocardial infarction, cardiothoracic surgery, stroke, and trauma) [1–7]. However, its prevalence is difficult to ascertain due to the absence in early papers of a universally accepted definition of SH, inhomogeneity of study populations, differences in severity of illness, divergent ways of reporting blood glucose readings, and varied timing and frequency of blood glucose sampling [8]. Contributing factors leading to SH include inflammatory mediators, excessive release of counter-regulatory hormones, insulin resistance and medical interventions (e.g., administration of corticosteroids, vasopressors, dextrose solutions, enteral or parenteral nutrition, and dialysis). SH is also related to the severity of the underlying illness or injury [9]. A patient’s predisposition (age, body mass index (BMI), family history of diabetes, beta cell reserve) may also play an important role in the development of SH.

Although strict glycaemic control (80–110 mg/dl) is no longer advocated for most ICUs, there is a consensus that manifest hyperglycaemia should be treated and insulin-induced hypoglycaemia should be avoided [7, 10, 11]. The Society of Critical Care Medicine has recently published new guidelines that recommend a target range of 100–150 mg/dl [12].

Few studies, however, provide insight into the long-term follow up after SH (>140 mg/dl). In one prospective single-centre study, patients admitted to a medical ICU were screened post-discharge by an annual 75 g oral glucose tolerance test (OGTT) during a five-year follow-up period [13]. 17.1 % of the critically ill patients with documented SH and normal post-discharge glucose tolerance developed diabetes mellitus, versus 3.5 % of subjects who were normoglycaemic during their ICU stay. Prompt recognition of a disturbed glucose metabolism or diabetes mellitus would lead to optimal therapeutic management.

We speculate that SH in patients without prior diagnosis of diabetes could be a manifestation of a latent disturbance in the glucose metabolism. Our primary objective was to determine the incidence of a disturbed glucose metabolism six to nine months post-ICU admission. We also aimed to identify predictors of future diabetes risk.

## Methods

### Setting and participants

**Fig. 1:**
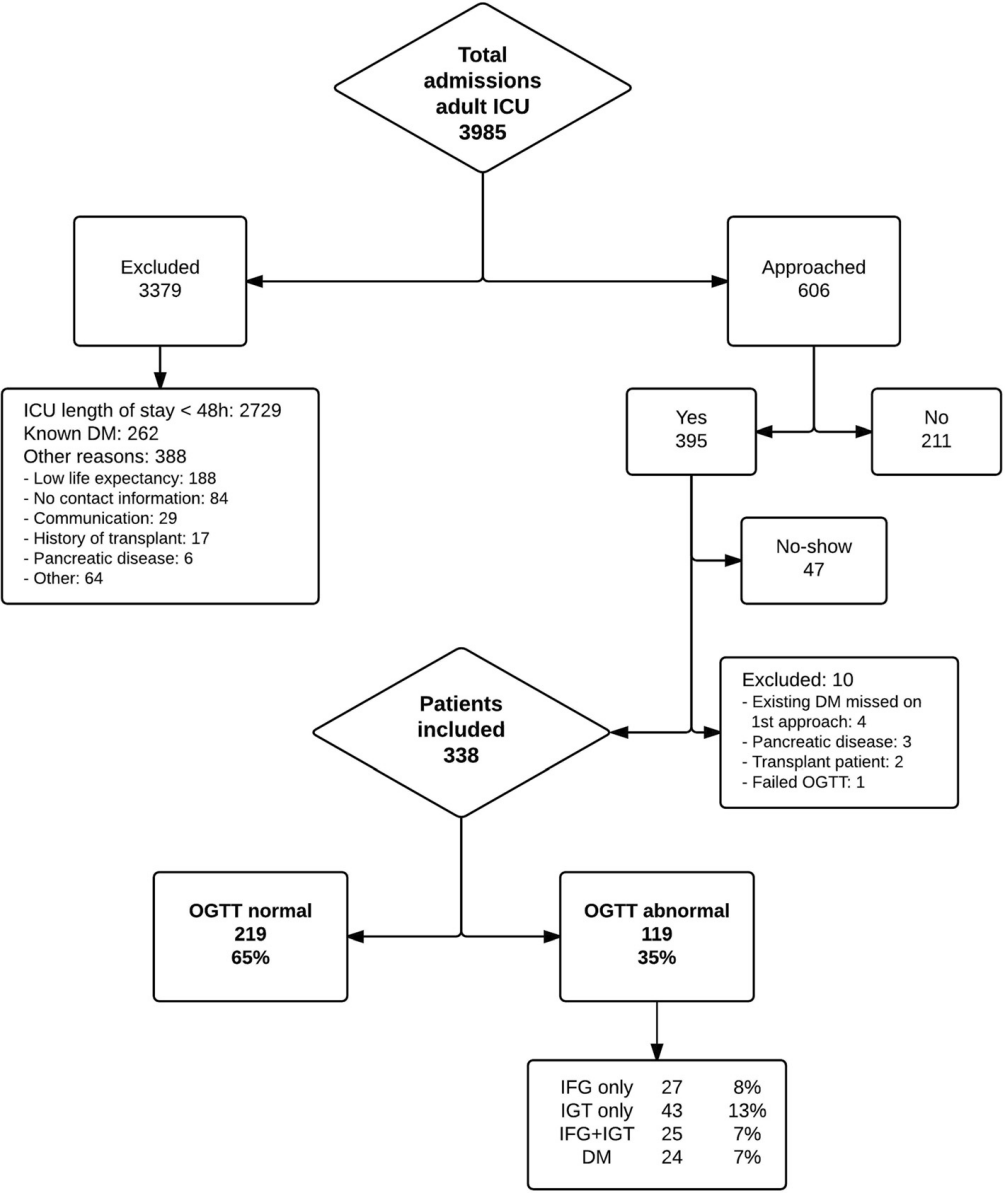
Overview of the inclusion process and glucose metabolism status. *OGTT* oral glucose tolerance test, *IFG* impaired fasting glucose, *IGT* impaired glucose tolerance, *DM* diabetes mellitus type 2

This prospective, observational study was performed at the Antwerp University Hospital, Edegem, Belgium. During the study period between September 2011 and March 2013, a total of 3,985 adult critically ill patients were admitted to the 45-bed mixed medical-surgical ICU (nurse-to-patient ratio 1:2.5 to 1:3.0). Only adult patients aged between 18 and 85 years admitted for 48 h or longer to the ICU and who were still alive six months thereafter were eligible, leaving a total of 1,256 subjects. Subjects with known diabetes or any other glucose metabolism disturbance (impaired fasting glucose and/or impaired glucose tolerance) and individuals using glucose-lowering drugs were excluded from participation in this study (n = 262). Other exclusion criteria included estimated short life expectancy, pregnancy and/or a history of transplantation or acute pancreatic disease. Of the 395 subjects who agreed to participate, 47 did not show up for the OGTT, leaving 348 subjects, of whom another 10 were excluded, 4 of whom had existing disturbed glucose metabolism (treated by oral antidiabetic agents or insulin) and were missed on first approach. This resulted in 338 subjects being included in the study. These numbers can be found in Fig. 1.

### Glucose control

Blood glucose levels were drawn from an arterial line and measured using an on-site blood gas analyser (Rapidlab® 1265, Siemens, München, Germany) and they were used to adjust the insulin infusion rate. Insulin aspart (Novo Nordisk, Bagsvaerd, Denmark), in a concentration of 50 units in 50 cc 0.9 % NaCl, was continuously infused using the Injectomat Agilia® syringe infusion system (flow-rate change, range 0.1−200 ml/h, Fresenius Kabi, Bad Homburg, Germany). During the ICU stay, treatment of hyperglycaemia was performed using the adapted Yale protocol, maintaining glycaemia between 60 and 140 mg/dl [14]. SH was defined as glucose levels exceeding this threshold of 140 mg/dl, in accordance with the consensus statement [15]. Patients were fed according to the local ICU policy, preferably enterally.

### Data elements collected

Anthropometric data (length, weight, BMI, waist circumference), preadmission home medication, reason for ICU admission, parameters of disease severity including the simplified acute physiology score (SAPS 3), [16, 17] the daily sequential organ failure assessment score (SOFA score), [18] and length of stay in hospital and in the ICU, treatment during ICU stay (steroids, vasopressors, inotropes, mechanical ventilation, insulin), total caloric intake (based on total parenteral nutrition (TPN), intravenous (IV) fluids and enteral feeds but without propofol-associated calories), and laboratory values (C-reactive protein and lactate levels) were extracted from the electronic patient records. The following glucose parameters were analysed in accordance with the consensus report on glucometrics: maximum and mean glycaemia at day of admission and at ICU discharge, HbA1c on admission, mean insulin dose per day, percentage of glucose values in target range (60–140 mg/dl), mean glycaemia during ICU stay, and standard deviation of glycaemia in individual patients) [8].

### Outcomes and follow up

An outpatient visit was planned approximately six months after ICU discharge. At the moment of this appointment, the Finnish diabetes risk score (FINDRISC) questionnaire (containing age, BMI, waist circumference, diet, exercise, history of hypertension and personal and family history of elevated blood glucose) was presented [19]. We also inquired about smoking status and home medication, and length, weight and waist circumference were measured at that time.

A 2-h OGTT with a 75-g glucose load was performed according to the World Health Organisation (WHO) guidelines [20, 21]. The following definitions were used for interpretation of the OGTT result [20]: diabetes mellitus – fasting plasma glucose ≥126 mg/dl and/or 2-h plasma glucose during the OGTT ≥200 mg/dl and/or HbA1c ≥6.5 % (48 mmol/mol); impaired glucose tolerance – 2-h plasma glucose during the OGTT ≥140 mg/dl but <200 mg/dl. Impaired fasting glucose was defined as fasting plasma glucose ≥100 mg/dl and <126 mg/dl, according to the American Diabetes Association guidelines [20].

### Statistical analysis

The data are expressed as mean (standard deviation) for normally distributed continuous variables and as median (range) for non-normally distributed continuous variables. Categorical data are expressed as number (percent). The normal distribution of continuous variables was assessed with the Kolmogorov-Smirnov method. The unpaired *t* test and the Mann–Whitney *U* test were used to compare means and medians, respectively. For polychotomous outcome parameters, we used one-way analysis of variance (ANOVA) with the Bonferroni correction or Kruskall-Wallis ANOVA with a Tamhane T2 test. A contingency table was generated to assess potential significant differences between the groups in categorical variables, and Fisher’s exact test was applied. Based on clinical judgement and after univariate analysis, we entered all parameters that could likely be associated with disturbed glucose metabolism in a logistic regression model, and assessed the corrected effect of those elements using bootstrapping. Odds ratios were converted to risk ratios. A two-tailed *P* value <0.05 was considered significant. Statistical analyses were performed with SPSS Statistics software, version 20.0 for Mac (Armonk, NY, USA).

### Ethics

This study was conducted in accordance with the amended Declaration of Helsinki. The research protocol was approved by the University of Antwerp and Antwerp University Hospital Ethics committee (registration number EC 12/2/22) prior to initiation of the study. All participants gave signed informed consent.

## Results

### Population characteristics

The study population consisted of 338 patients (of whom 223 were male); 326 (96 %) were Caucasian. The HbA1c on admission was measured in 151 patients (45 % of the total study population) and was <6.5 % in all patients included for analysis. A total of 246 patients (73 %) had received insulin therapy during their ICU admission and were therefore labelled as having experienced SH. The mean interval between ICU admission and time of OGTT was eight months, with a 10−90 percentile range of seven to ten months.

### Characteristics comparing the two groups with normal and disturbed glucose metabolism

**Fig. 2:**
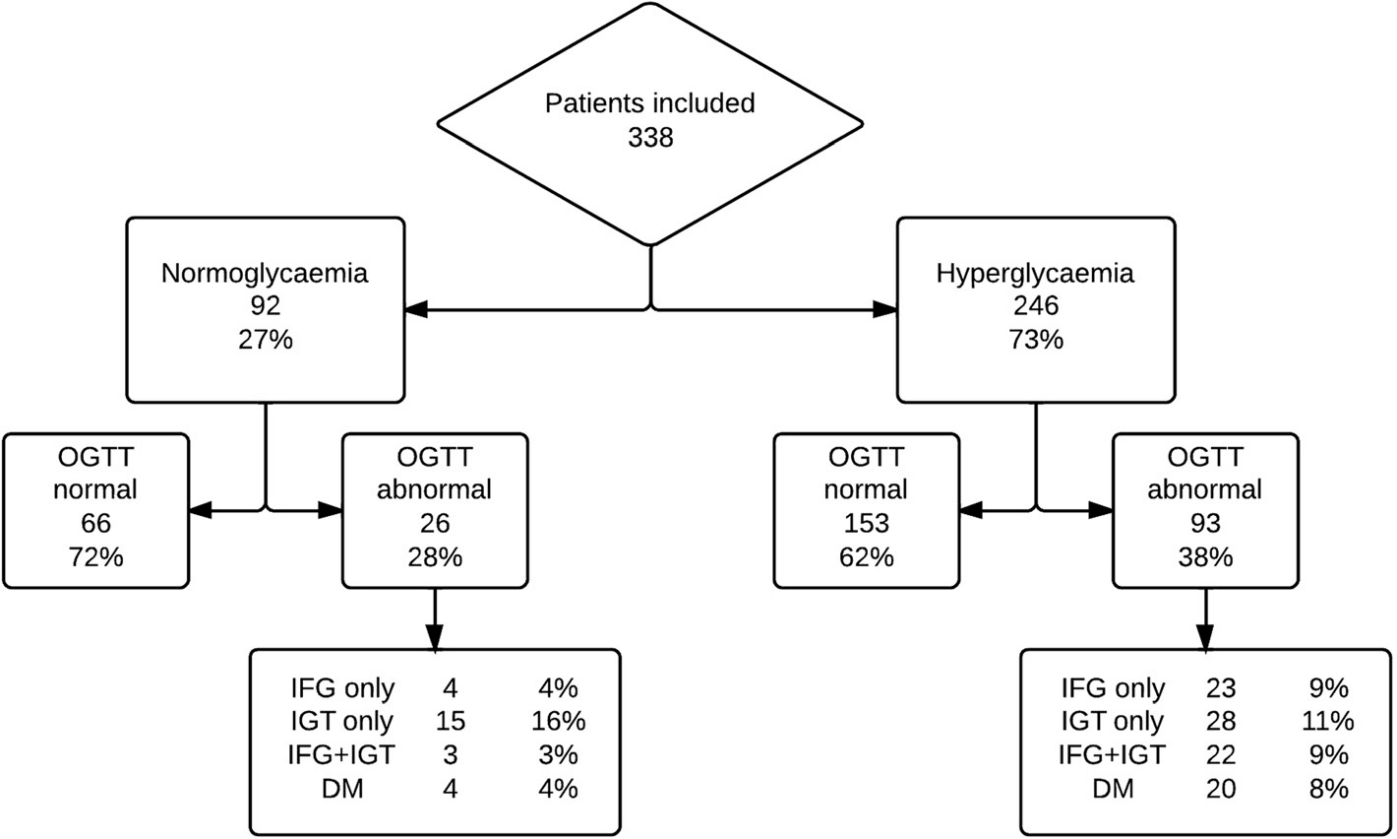
Glucose metabolism status of patients with and without stress hyperglycaemia during ICU admission. *OGTT* oral glucose tolerance test, *IFG* impaired fasting glucose, *IGT* impaired glucose tolerance, *DM* diabetes mellitus type 2

A total of 119 patients (35 %) had an abnormal OGTT result; 27 patients (8 %) had impaired fasting glucose only, 43 (13 %) had impaired glucose tolerance only, and 25 (7 %) had both impaired fasting glucose and impaired glucose tolerance; in 24 (7 %) the criteria for diabetes mellitus were met (Fig. 1). Among the 24 patients diagnosed with diabetes, only 5 had an HbA1c ≥6.5 % at the time of the OGTT, indicating that the diagnosis was made in the early stages of the disease for most diabetes patients. On the other hand, 1 out of 24 patients diagnosed with diabetes on the basis of an HbA1c ≥6.5 % had an OGTT not indicating diabetes, and would have been misclassified when only performing an OGTT. Patients who had experienced SH during their ICU stay tended to be more frequently affected by disturbed glucose metabolism (38 % vs. 28 %, *P* = 0.065) and diabetes (9 % vs. 4 %, *P* = 0.246) than the normoglycaemic group (Fig. 2).

Table 1 compares baseline characteristics, disease severity parameters, metabolic parameters and FINDRISC score in subjects with normal glucose metabolism and those with disturbed glucose metabolism. Subjects admitted to the ICU after surgery tended to be more prone to develop disturbed glucose metabolism than medical ICU patients (*P* = 0.051). Most parameters of disease severity (SAPS 3 and SOFA score, mechanical ventilation and length of stay at the hospital and in the ICU) were comparable between the two groups. Those with disturbed glucose metabolism were not more severely ill at the time of their ICU stay than those with normal glucose tolerance. Vasopressor and inotropic therapy, but not corticosteroid treatment was more frequently administered in the group that later developed disturbed glucose metabolism.

**Table 1:** Parameters of patients with normal and disturbed glucose metabolism

	Normal	DGM	*P* value
Group size	(n = 219)	(n = 119)	
Baseline characteristics			
Sex			
Men	131 (59.8 %)	92 (77.3 %)	0.001
Women	88 (40.2 %)	27 (22.7 %)	
Age, years	57 (18–88)	67 (29–83)	<0.001
Weight, kg	75 ± 14	80 ± 16	0.003
Body mass index, kg/m^2^	25 (16–41)	26 (18–41)	0.002
Waist, cm	93 (66–124)	99 (66–133)	<0.001
Reason for admission			
Medical	84 (38.4 %)	33 (27.7 %)	NS
Surgical	135 (61.6 %)	86 (72.3 %)	
Primarily involved organ system			
Cardiovascular	108 (49.3 %)	71 (59.7 %)	NS
Respiratory	46 (21.0 %)	20 (16.8 %)	NS
Digestive	16 (7.3 %)	6 (5.0 %)	NS
Renal	4 (1.8 %)	3 (2.5 %	NS
Hepatic	8 (3.7 %)	3 (2.5 %)	NS
Haematologic	2 (0.9 %)	0 (0.0 %)	NS
Neurologic	53 (24.2 %)	19 (16.0 %)	NS
Disease severity			
Simplified acute physiology score III	42 (16–90)	42 (23–85)	NS
SOFA score on admission	8 (1–18)	8 (2–19)	NS
Maximum SOFA score during admission	8 (2–20)	8 (2–19)	NS
Vasopressor/inotropic therapy	120 (54.8 %)	79 (66.4 %)	0.039
Corticosteroid therapy	35 (16.0 %)	12 (10.1 %)	NS
Mechanical ventilation	134 (61.2 %)	73 (61.3 %)	NS
Length of stay at the ICU, hours	95 (39–1416)	94 (37–503)	NS
Length of stay at the hospital, days	15 (3–163)	14 (2–86)	NS
Metabolic parameters			
Mean glycaemia, day 1, mg/dl	130 (83–469)	130 (86–232)	NS
SD glycaemia, day 1	22 (1–615)	22 (4–168)	NS
Maximum glycaemia, day 1, mg/dl	168 (87–591)	166 (86–600)	NS
Number of patients with hypoglycaemic event, day 1	11 (5.1 %)	7 (5.9 %)	NS
glucose values in target, 60–140 mg/dl (%, range)	70 (0–100)	67 (14–100)	NS
Mean (SD) glycaemia, mg/dl	128 (18)	131 (27)	NS
Median (IQR) glycaemia, mg/dl	129 (120–137)	130 (117–140)	NS
SD glycaemia levels in individual patients, mg/dl, median (range)	27 (19–34)	30 (21–40)	NS
HbA1c on admission, (%)	5.5 (4.7–6.2)	5.6 (5.0–6.4)	0.025
HbA1c on admission, (mmol/mol)	37 (28–44)	38 (31–46)	
Insulin therapy	153 (69.9 %)	93 (78.2 %)	NS
Mean insulin dose, units/day	9 (0–100)	12 (0–99)	0.048
Total parental nutrition	15 (6.8 %)	1 (0.8 %)	0.013
Caloric intake, kcal/day	222 (0–2127)	197 (32–1911)	0.013
ICU exit parameters			
C-reactive protein, last day, mg/l	5.7 (0.0-30.0)	8.1 (0.0-29.0)	0.015
Lactate, last day, mmol/l	1.3 (0.4-79.0)	1.5 (0.7-4.0)	0.006
Mean glucose last day, mg/dl	115 (84–164)	122 (82–217)	<0.001
Maximum glucose, last day, mg/dl	136 (91–438)	157 (94–636)	0.001
FINDRISC score	9.5 (0–20)	11 (3–21)	<0.001
FINDRISC score ≥14	30 (14.3 %)	33 (28.2 %)	0.003
HbA1c upon OGTT, (%)	5.4 (4.3-6.1)	5.6 (4.9-8.4)	<0.001
HbA1c upon OGTT, (mmol/mol)	36 (23–43)	38 (30–68)	
HbA1c ≥6.5 % upon OGTT	0	5 (4.2 %)	0.005

Numbers are mean ± SD for continuous, normally distributed data, median (range) for continuous, not normally distributed data, and absolute and relative numbers for discontinuous data. Day 1 = first 24 h of admission. Hypoglycaemic event = 1 glucose value <60 mg/dl. *P* value was deemed significant when <0.05. *DGM* disturbed glucose metabolism, *NS* non-significant, *SOFA* sequential organ failure assessment, *FINDRISC* Finnish diabetes risk score, *HbA1c* glycated haemoglobin, *OGTT* oral glucose tolerance test

Important differences in metabolic parameters were observed between the two groups during their stay at the ICU. Patients with disturbed glucose metabolism had a higher HbA1c on ICU admission (*P* = 0.025) and required a higher mean insulin dose per day (*P* = 0.048). Additionally, fewer patients received TPN in this group (1 vs. 15 subjects, *P* = 0.013) and the caloric intake was statistically significantly lower (*P* = 0.013). However, the percentage of glucose values within target, mean glycaemia and standard deviation of glycaemia in individual patients did not differ significantly between the normal and the disturbed glucose metabolism (DGM) group.

Several metabolic parameters on the last day of ICU stay (ICU exit parameters) were higher in patients with disturbed glucose metabolism (CRP, lactate, mean and maximum glucose), indicating that these patients had more severe metabolic disturbances upon discharge.

The FINDRISC score was significantly higher in the group with disturbed glucose metabolism, with 28.2 % of patients having a FINDRISC score ≥14, compared to 14.3 % in the group of patients with normal glucose metabolism (*P* = 0.003). As expected, the same was true for the HbA1c measured during the OGTT (*P* <0.001).

### Independent predictors for disturbed glucose metabolism after ICU stay

We further investigated the possible predictors of disturbed glucose metabolism through multivariate analysis, incorporating clinically important parameters that were significantly correlated in univariate analysis: FINDRISC score, HbA1c on admission, calories per day and relevant laboratory values on the last day (CRP, lactate). The baseline anthropometric characteristics are already integrated in the FINDRISC score. The SAPS III score, the presence of SH and the use of steroid drugs during ICU stay were included as well, likely being clinically relevant but not significantly associated in the univariate analysis. The FINDRISC score (*P* = 0.001), the SAPS III score (*P* = 0.003) and the mean caloric intake per day (*P* = 0.011) were associated with disturbed glucose metabolism in this multivariate analysis. Patients with a FINDRISC score ≥14 had more than a 50 % chance of developing disturbed glucose metabolism. When excluding the FINDRISC score from the multivariate analysis, the presence of SH was predictive of future disturbed glucose metabolism as well (relative risk (RR)  = 2.221, 95 % CI 1.201, 2.995, *P* = 0.033). The median caloric intake per day was marginally lower (197 vs. 222 kcal/day, *P* = 0.021) in the group with disturbed glucose metabolism.

### The value of the HbA1c concentration on admission

**Fig. 3:**
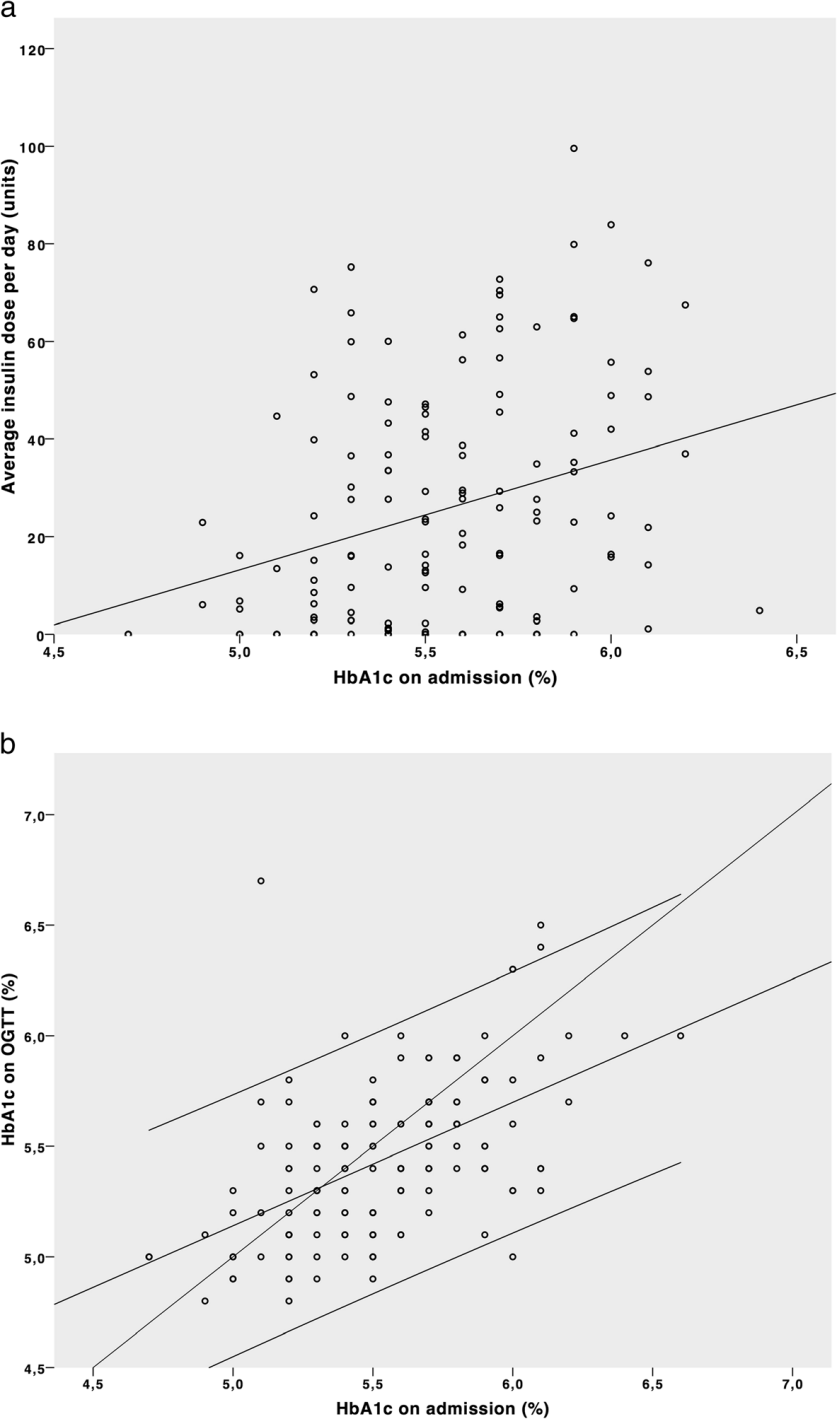
**a** Correlation between glycated haemoglobin (*HbA1c*) on admission and need for insulin during ICU admission. **b** Relation between HbA1c on admission and upon oral glucose tolerance test (OGTT) after 8 months. HbA1c is expressed as percent. Three *parallel lines* represent the mean and 95 % CI; the *fourth and steepest line* is a line connecting equal values on the x and y axis. The values that are located under this fourth line have decreased (improved), while those above the line have increased (deteriorated)

A higher HbA1c level on admission, even when still within the normal range, was associated with a higher insulin need during the ICU stay (*r* = 0.308, *P* <0.001, Fig. 3a), and with a higher risk of disturbed glucose metabolism afterwards, albeit only in univariate analysis.

In addition, subjects with a higher HbA1c at the time of ICU admission also displayed a higher HbA1c eight months later (*r* = 0.552, *P* <0.001, Fig. 3b).

### Characteristics comparing the two groups with and without stress hyperglycaemia

Table 2 compares patients with and without SH during ICU admission. SH was more prevalent in surgical patients as compared to medical ICU patients. SH was most commonly found in patients admitted for cardiovascular reasons. Patients with SH were more severely ill as demonstrated by a higher SOFA score, more vasopressor and/or inotropy need, more need for mechanical ventilation, and a longer stay in ICU. The FINDRISC score was also higher in subjects with SH.

**Table 2:** Parameters of patients with and without stress hyperglycaemia

	Normal	SH	*P* value
Group size	(n = 92)	(n = 246)	
Baseline characteristics			
Sex			
Men	53 (57.6 %)	170 (69.1 %)	0.053/NS
Women	39 (42.4 %)	76 (30.9 %)	
Age, years	56 (18–82)	62 (20–88)	0.005
Weight, kg	74 ± 15	78 ± 15	0.027
Body mass index, kg/m^2^	25 (16–41)	26 (17–41)	0.006
Waist, cm	93 (66–128)	96 (66–133)	0.030
Reason for admission			
Medical	52 (56.5.4 %)	65 (26.4 %)	<0.001
Surgical	40 (43.5 %)	181 (73.6 %)	
Primarily involved organ system			
Cardiovascular	30 (32.6 %)	149 (60.6 %)	<0.001
Respiratory	22 (23.9 %)	44 (27.9 %)	NS
Digestive	7 (7.6 %)	15 (6.1 %)	NS
Renal	0 (0.0 %)	7 (2.8 %)	NS
Hepatic	5 (5.4 %)	6 (2.4 %)	NS
Haematologic	1 (1.1 %)	1 (0.4 %)	NS
Neurologic	32 (34.8 %)	40 (16.3 %)	0.001
Disease severity			
Simplified acute physiology score III	41 (16–76)	42 (18–90)	NS
SOFA score on admission	5 (2–16)	8 (1–19)	<0.001
Maximum SOFA score during admission	6 (2–16)	8 (2–20)	<0.001
Vasopressor/inotropic therapy	18 (19.6 %)	181 (73.6 %)	<0.001
Corticosteroid therapy	11 (12.0 %)	36 (14.6 %)	NS
Mechanical ventilation	20 (21.7 %)	187 (76.0 %)	<0.001
Length of stay in the ICU, hours	82 (39–1075)	99 (37–503)	<0.001
Length of stay in hospital, days	14 (2–163)	3 (3–115)	NS
Metabolic parameters			
Mean glycaemia, day 1, mg/dl	121 (87–217)	134 (86–232)	<0.001
SD glycaemia, day 1	13 (1–99)	26 (5–168)	<0.001
Maximum glycaemia, day 1, mg/dl	137 (87–348)	176 (91–600)	<0.001
Number of patients with hypoglycaemic event, day 1	1 (1.1 %)	17 (6.9 %)	NS
HbA1c on admission, (%)	5.4 (4.7–5.9)	5.5 (4.9–6.6)	0.004
HbA1c on admission, mmol/mol	36 (28–41)	37 (30–49)	
Total parental nutrition	15 (6.8 %)	1 (0.8 %)	0.013
Caloric intake, kcal/day	214 (29–2048)	206 (0–2127)	NS
ICU exit parameters			
C-reactive protein, last day, mg/l	5.4 (0.0-30.0)	7.1 (0.0-29.0)	0.025
Lactate, last day, mmol/l	1.0 (0.4-79.0)	1.5 (0.4-21.2)	<0.001
Mean glucose last day, mg/dl	111 (87–217)	119 (82–209)	<0.001
Maximum glucose, last day, mg/dl	127 (94–296)	148 (91–636)	<0.001
FINDRISC score	9.0 (0–21)	10.0 (0–21)	0.001
FINDRISC score ≥14	11 (12.6 %)	52 (21.7 %)	NS
HbA1c upon OGTT (%) (mmol/mol)	5.4 (4.3-8.4)	5.5 (4.8-6.7)	0.001
HbA1c upon OGTT, mmol/mol	35 (24–68)	37 (29–50)	
HbA1c ≥6.5 % upon OGTT	2 (2.2 %)	3 (1.2 %)	NS

Numbers are mean ± SD for continuous, normally distributed data, median (range) for continuous, not normally distributed data, and absolute and relative numbers for discontinuous data. Day 1 = first 24 h of admission. Hypoglycaemic event = 1 glucose value <60 mg/dl. *P* value was deemed significant when <0.05. *DGM* disturbed glucose metabolism, *NS* non-significant, *SOFA* sequential organ failure assessment, *FINDRISC* Finnish diabetes risk score, *HbA1c* glycated haemoglobin

## Discussion

This prospective analysis is one of the largest and most detailed to date to study the relationship between SH and post ICU glucose tolerance in a mixed ICU population without known diabetes. SH is frequent in non-diabetic ICU patients, but whether this is a manifestation of a latent disturbance in the glucose metabolism, and puts an individual at a higher risk of future diabetes remains unclear [13, 22–24]. Glucose metabolism was disturbed in 35 % of patients post ICU admission, of whom 7 % were diagnosed with diabetes. We are, to the best of our knowledge, the first to explore this in a mixed ICU population (medical and surgical patients) and to identify the SAPS III score as a predictor of disturbed glucose metabolism after an ICU stay. We also observed that a higher FINDRISC score is a valid tool to screen for subjects at high risk of developing disturbed glucose metabolism or diabetes in this population.

### Incidence of disturbed glucose metabolism in comparison with previous data

Only one other study prospectively screened patients previously admitted to an ICU [13]. Performing an annual 75-g OGTT during a five-year follow-up period, the authors observed that 17.1 % of the critically ill patients with documented SH and normal post-discharge glucose tolerance developed diabetes, versus 3.5 % of subjects who were normoglycaemic during their ICU stay. However, a direct comparison with our study is difficult. The subjects in this study had a considerably longer follow-up period (five years). Although it provides an interesting long-term perspective, this automatically results in greater age and therefore higher incidence of disturbed glucose metabolism. In addition, the authors did not measure the HbA1c levels on admission and may have under-diagnosed diabetes at the time of ICU admission. Another consideration is the fact that we, unlike Gornik et al., followed a strict insulin treatment protocol in our ICU, thereby avoiding severe derailing of the glucose metabolism.

McAllister et al. performed a retrospective study in a large Scottish cohort, demonstrating that plasma glucose measured during emergency hospital admission predicted subsequent risk of developing diabetes type 2 [25]. The three-year risk was <1 % when admission glycaemia was ≤90 mg/dl, increasing to 2.6 % at 126 mg/dl, to 9.9 % at 200 mg/dl, and to approximately 15 % at 270 mg/dl. However, the only glucometric data reported in that study were for admission glucose, and the authors could not state whether these glucose values were taken in fasting or non-fasting conditions. We performed a broader analysis of glucometric data but did not observe significant differences between the normal and disturbed glucose metabolism group in the percentage of glucose values within target, mean glycaemia or SD of glycaemia in individual patients during their ICU stay. In contrast, glucose values on the last day of the ICU stay were significantly higher in those subjects developing disturbed glucose metabolism.

### Identifying predicting factors for disturbed glucose metabolism

Diabetes mellitus is a growing health problem worldwide and is reaching epidemic proportions [26]. Given the major implications following the diagnosis of disturbed glucose metabolism or diabetes and the potential benefits of an early adjustment of lifestyle and treatment, it is important to identify people who are at risk of developing diabetes.

In this study, the FINDRISC score and SAPS III on admission were positively correlated with disturbed glucose metabolism and mean caloric intake during the ICU stay was negatively correlated with disturbed glucose metabolism. Disturbed glucose metabolism also tended to be more prevalent in patients who experienced SH during the ICU stay as compared to those without SH. Patients with SH were more severely ill as demonstrated by a higher SOFA score, more vasopressor and/or inotropy need, more need for mechanical ventilation, and a longer stay in the ICU. The difference in length of stay can be considered as a confounder taking the more frequent blood glucose measurements into account, which are performed when patients stay longer in the ICU. However, the degree of SH, reflected by the mean insulin dose per day was significantly correlated with the OGTT result. In the study by Gornik et al., patients with a positive family history of diabetes and with a higher BMI were also more prone to develop diabetes [13]. Both factors are included in the FINDRISC score.

The FINDRISC score was higher in those with SH during ICU admission. We suspect that SH might be a manifestation of a latent disturbance in the glucose metabolism. Of all investigated parameters, background clinical characteristics of patients (such as those incorporated in the FINDRISC score) were most strongly associated with the future development of DM or disturbed glucose metabolism. Patients with a higher FINDRISC score thus seem to be more likely to have disturbed glucose tolerance after and during their ICU stay, as reflected by the development of SH. Our results show a 10 % increased risk of SH with a one-point increase in the FINDRISC score. The underlying physiological rationale is most likely to be insulin resistance, as several components of the FINDRISC score, such as age, BMI, waist circumference, lack of exercise, history of hypertension and personal and family history of elevated blood glucose are associated with insulin resistance.

The HbA1c concentration on admission correlated with a disturbed glucose metabolism after hospital discharge. Other studies in non-ICU settings such as acute stroke and myocardial infarction came to the same conclusion [24, 27–29]. Furthermore, we observed an association between the HbA1c concentration on admission and the degree of SH being reflected by the mean insulin dose per day. Considering all patients had an HbA1c <6.5 % on admission, we can argue that even when within the normal range, elevated HbA1c indicates a predisposition to SH, demonstrating a pre-existing tendency towards glucose metabolism disturbances including diabetes. These findings are in agreement with those of other studies, which concluded that above a certain threshold of disease severity the degree of SH is no longer solely determined by the severity of illness itself; instead, it can be determined by the underlying reserve of the glucose metabolism [24, 30]. The HbA1c on admission reflects this reserve. These findings support our hypothesis that SH is an expression of latent xdisturbance of glucose metabolism rather than a causative mechanism. The fact that the disease severity characteristics and interventions during the ICU stay (including corticosteroid treatment) did not correlate with the OGTT result in the univariate analysis further supports this hypothesis.

The median caloric intake was only approximately 200 kcal per day, with only 25 kcal per day difference between those with normal and disturbed glucose metabolism. It is unlikely that this relatively small difference is of clinical relevance. Caloric intake was low because 65 % of the study population consisted of surgical patients and the policy of *nil per o*s on the first day after surgery.

### Limitations

In our study we only included adult patients admitted for ≥48 h to the ICU. Based on the exclusion criteria, the number of patients who were eligible to participate was relatively small, making it difficult to generalize the results. However, determining the true prevalence of diabetes mellitus and disturbed glucose metabolism in critically ill patients is difficult. To exclude patients with a history of disturbed glucose metabolism, we used the medical file records and the HbA1c on admission. Although HbA1c values are considered superior to the OGTT and fasting plasma glucose as a diagnostic tool during hospital admission, not all diabetes patients have an HbA1c ≥6.5 % in the early stages of the disease [20, 31]. However, a large proportion of patients might still bear the effects of SH when an OGTT is performed too soon after admission, which might lead to false positive results and over-diagnosis.

The FINDRISC score was an efficient predictor of OGTT abnormalities. This score was calculated upon the execution of the OGTT, i.e., approximately eight months after ICU admission. A number of patients may have implemented lifestyle changes after they were admitted to the ICU. Therefore, theoretically there could be a difference if the FINDRISC score is calculated before hospital discharge.

Due to the nature of our study in which patients actively had to visit the hospital for the OGTT, those patients who remained severely ill or were less mobile (and therefore might run a higher risk of disturbed glucose metabolism or diabetes) may not have been able to participate in the study. This probably induces a negative bias, underestimating the prevalence of disturbed glucose metabolism.

## Conclusions

We observed disturbed glucose metabolism in one out of three patients approximately eight months after ICU admission. SH during the ICU stay might be an expression of latent impairment of glucose metabolism, which is demonstrated by the tendency towards disturbed glucose metabolism and diabetes mellitus after discharge. However, the fact that not only those who experienced SH, but also those who remained normoglycaemic during their ICU stay developed disturbed glucose metabolism, emphasizes the fact that SH is not the only factor that predicts subsequent development of disturbed glucose metabolism. Clinically relevant factors associated with disturbed glucose metabolism in this study are a high FINDRISC score and a higher SAPS III on admission. These predictors can provide an efficient, quick and inexpensive way to identify patients at risk of disturbed glucose metabolism or diabetes. Systematic screening and prompt recognition of disturbed glucose metabolism or diabetes may lead to optimised therapeutic management.

## Key messages

- HbA1c on ICU admission correlates with the degree of ICU-related stress hyperglycaemia
- Eight months post ICU admission (for ≥36 h), 35 % of subjects have disturbed glucose metabolism
- Eight months post ICU admission (for ≥ 36 h), 7 % of patients are diagnosed with diabetes mellitus
- Disturbed glucose metabolism post ICU admission tended to be more prevalent in patients who experienced stress hyperglycaemia during their ICU stay
- A validated diabetes risk questionnaire (FINDRISC) and SAPS III provide an efficient way to identify patients at risk of disturbed glucose metabolism after ICU discharge

## Abbreviations

BMI: body mass index
CRP: C-reactive protein
DGM: disturbed glucose metabolism
FINDRISC: Finnish diabetes risk score
HbA1c: glycated haemoglobin
ICU: intensive care unit
IFG: impaired fasting glucose
IGT: impaired glucose tolerance
OGTT: oral glucose tolerance test
SAPS III: simplified acute physiology score
SH: stress hyperglycaemia
SOFA: sequential organ failure assessment score
TPN: total parenteral nutrition
WHO: World Health Organisation
